# Using *Drosophila* to study mechanisms of hereditary hearing loss

**DOI:** 10.1242/dmm.031492

**Published:** 2018-05-31

**Authors:** Tongchao Li, Hugo J. Bellen, Andrew K. Groves

**Affiliations:** 1 Program in Developmental Biology, Baylor College of Medicine, Houston, TX 77030, USA; 2 Jan and Dan Duncan Neurological Research Institute, Texas Children's Hospital, Houston, TX 77030, USA; 3 Department of Molecular and Human Genetics, Baylor College of Medicine, Houston, TX 77030, USA; 4 Howard Hughes Medical Institute, Baylor College of Medicine, Houston, TX 77030, USA; 5 Department of Neuroscience, Baylor College of Medicine, Houston, TX 77030, USA

**Keywords:** Cochlea, Deafness, *Drosophila*, Hair cells, Hearing, Usher syndrome

## Abstract

Johnston's organ – the hearing organ of *Drosophila* – has a very different structure and morphology to that of the hearing organs of vertebrates. Nevertheless, it is becoming clear that vertebrate and invertebrate auditory organs share many physiological, molecular and genetic similarities. Here, we compare the molecular and cellular features of hearing organs in *Drosophila* with those of vertebrates, and discuss recent evidence concerning the functional conservation of Usher proteins between flies and mammals. Mutations in Usher genes cause Usher syndrome, the leading cause of human deafness and blindness. In *Drosophila*, some Usher syndrome proteins appear to physically interact in protein complexes that are similar to those described in mammals. This functional conservation highlights a rational role for *Drosophila* as a model for studying hearing, and for investigating the evolution of auditory organs, with the aim of advancing our understanding of the genes that regulate human hearing and the pathogenic mechanisms that lead to deafness.

## Abstract (precis)

**Summary:** This Review discusses the similarities and differences between hearing in mammals and the fruit fly *Drosophila*, and describes how recent technological developments allow *Drosophila* to be used as a model to understand the function of human deafness genes.

## Introduction

Hearing loss is one of the most common neurological disorders in humans, affecting 2 to 3 of every 1000 children in the USA (Imtiaz et al., 2016; Naz et al., 2017). Approximately 50% of congenital hearing loss is caused by genetic mutations, with a variety of other factors, particularly infectious disease, contributing to the remaining forms of congenital deafness (Korver et al., 2017). Genetic mutations affect many components of the auditory pathway, and can affect only hearing (nonsyndromic deafness), or other organs and tissues as well (syndromic deafness). Of the genetic mutations that cause nonsyndromic deafness, ∼30% are autosomal dominant, ∼70% are autosomal recessive, and five are X-linked (hereditaryhearingloss.org). Among the many forms of syndromic hearing loss are Alport syndrome, Pendred syndrome, Branchio-oto-renal syndrome, Stickler syndrome and Usher syndrome (Korver et al., 2017). Usher syndrome is the leading cause of human deafness and blindness, with a prevalence of 1-4 per 25,000 people in the USA (Boughman et al., 1983; Hartong et al., 2006; Keats and Corey, 1999; Kimberling et al., 2010; Mathur and Yang, 2015). It is an autosomal recessive genetic disease, characterized by varying degrees of deafness and retinitis pigmentosa-induced vision loss. Although our understanding of genetic hearing loss has advanced greatly over the past 20 years (Vona et al., 2015), there is a pressing need for experimental systems to understand the function of the proteins encoded by deafness genes. The mouse is well established as a model for studying human genetic deafness (Brown et al., 2008), but other model organisms, such as the fruit fly *Drosophila*, might also provide convenient and more rapid ways to assay the function of candidate deafness genes.

In mammals, mechanosensitive hair cells reside in a specialized epithelial structure, the organ of Corti (see Glossary, Box 1), that runs along the length of the cochlear duct (Box 1) of the inner ear (Basch et al., 2016). Vibration of the organ of Corti in response to sound waves that are captured and amplified by the external and middle ears causes a deflection of the hair-like stereocilia of hair cells (Box 1). This deflection opens ion channels, leading to the release of neurotransmitters by hair cells and the activation of the first neurons in the auditory pathway (Fettiplace and Hackney, 2006). *Drosophila* also have a hearing organ – Johnston's organ (Box 1) – located in the antenna, the second segment of which contains several hundred mechanosensitive units called scolopidia (Eberl and Boekhoff-Falk, 2007). Bulk air displacements caused by near-field sound stimuli such as courtship song rotate the distal antennal segments, stretching the scolopidia (Boekhoff-Falk and Eberl, 2014). Accumulating evidence from *Drosophila* suggests that there are many physiological, molecular and genetic similarities between vertebrate and invertebrate auditory organs (Albert and Göpfert, 2015; Bokolia and Mishra, 2015; Jarman and Groves, 2013; Lu et al., 2009; Senthilan et al., 2012). For example, vertebrates and invertebrates share homologous transcription factors that specify the development of their sensory cells, the analogous accessory structures to capture and transduce sound energy, and the use of analogous systems to transduce and amplify mechanical force into electrical energy.

**Box 1. Glossary**

**Antennal segments:** the *Drosophila* antenna is made up of three segments: a1 (scape), a2 (or pedicelus), which contains Johnston's organ and responds to sound, gravity and wind flow, and a3 (or funiculus), the most distal segment, which is an olfactory organ.

**Arista:** a delicate, feather-like structure projecting from the third (most distal) antennal segment of *Drosophila* that is involved in sound detection.

**Chordotonal organs:** these organs are ciliated stretch receptors that detect motion in joints of the insect body. They are made up of repeating multicellular units called scolopidia (see below).

**Cochlea:** the coiled, snail shell-shaped part of the mammalian inner ear, the innermost portion of which, the cochlear duct, contains the organ of Corti (see below), which detects sound.

**CRISPR-mediated homology-directed repair:** the CRISPR-Cas9 system can be used to generate targeted double-stranded DNA breaks (DSBs) when coupled with a sequence-specific guide RNA (gRNA). When Cas9 and a gRNA are introduced into a cell with a piece of DNA flanked by homology arms, the DSB can be repaired by homologous recombination, inserting the new piece of DNA into the locus of interest.

**Forward genetic screens:** a genetic screen, typically of induced mutations, which identifies genes responsible for a particular phenotype, for example, hearing defects.

**GAL4-UAS system:** a two-component genetic system that activates gene expression in *Drosophila*. It uses the yeast GAL4 transcription factor to activate transcription of a transgene adjacent to an upstream activation sequence (UAS).

**Johnston's organ:** the largest chordotonal organ in *Drosophila*, containing ∼200 scolopidia. It is located in the second antennal segment and detects auditory signals (courtship songs), as well as wind flow and gravity.

**Lethal mosaic screen:** lethal homozygous mutations cannot be studied beyond the age they cause lethality. To circumvent this, clones of cells carrying two lethal mutant alleles can be generated in an otherwise heterozygous organism using a variety of recombination techniques. This approach allows researchers to assess the phenotypes caused by lethal mutations.

**Mechanotransduction channels:** ion channels that are gated by mechanical force.

**Organ of Corti:** an epithelial specialization of the cochlear duct, which detects sound through its mechanosensitive hair cells. The organ of Corti sits on a thin, flexible basilar membrane, which oscillates at different frequencies corresponding to its position along the cochlea.

**Recombination-mediated cassette exchange (RMCE):** regions of genomic DNA can be flanked by DNA sequences that act as targets for site-specific integrases. For example, the attB and attP DNA motifs are targets for the phiC31 integrase. If a piece of DNA bearing attB sites (a recombination cassette) is introduced into a cell that carries a DNA sequence flanked by attP sites in the presence of the phiC31 integrase, the intervening sequence will be replaced by the recombination cassette.

**Scolopidium:** a sensory unit of chordotonal organs, each containing 1-3 mechanosensitive sensory neurons, the cilia of which are surrounded by a scolopale cell. The scolopidium is anchored to the insect cuticle at its proximal and distal ends by a ligament cell and a cap cell, respectively. When flexional or rotational force is applied to the insect's cuticle, it is transferred to the sensory neurons in each scolopidium.

**Stereocilia:** elongated, actin-rich microvilli that protrude from the apical surface of sensory hair cells in vertebrates. Their displacement by force opens mechanosensitive ion channels, with their distal ends acting as likely sites of active mechanotransduction in hair cells. Rows of stereocilia of increasing length form a staircase-like bundle structure in hair cells, and the coupling of adjacent stereocilia by tip links transfers force to the entire bundle.

Here, we review the evidence that the genes, cells and molecular networks that specify the *Drosophila* hearing organ have mammalian counterparts that function in the differentiated cells of the cochlea and are required for hearing in mammals. In particular, we focus on recent findings that Usher syndrome proteins in *Drosophila* appear to physically interact in protein complexes that are similar to those described in mammals. We also describe recent work that indicates that some Usher syndrome proteins interact physically with Myosin II, the variants of which can cause *MYH9*-related diseases, including deafness. Various approaches, including forward genetic screens in *Drosophila* (Box 1) to identify mutants with behavioral defects associated with mechanosensory transduction (Eberl et al., 1997; Kernan et al., 1994), and microarray screens to identify transcripts enriched in Johnston's organ and regulated by *atonal*, have revealed striking similarities between the genes that are implicated in human deafness and those that affect the function and development of Johnston's organ in *Drosophila*. We also review the recently developed genetic toolkits in *Drosophila* that can characterize the function of novel genes in *Drosophila* hearing*.* It is estimated that several hundred genes involved in hearing still await discovery on the basis of mouse deafness mutants that have been isolated in mutagenesis and knockout screens (Steel, 2014). We suggest that by improving our understanding of the mechanisms of hearing in *Drosophila*, we could discover new genes required for hair cell function in vertebrates, better understand the evolution of auditory organs, and gain new insights into the pathogenic mechanisms of some deafness-related genes.

## Functional similarities between *Drosophila* and mammalian hearing organs

**Fig. 1.**
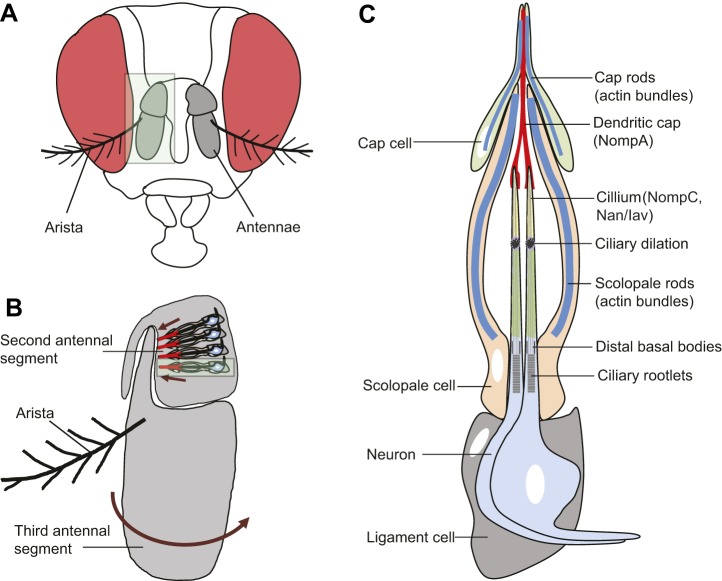
**Structure of the *Drosophila* auditory organ.** (A) A frontal view of an adult *Drosophila* head, with antennae marked in gray (eyes are in red). (B) Magnified image of an antenna (area highlighted in light green in A). The auditory organ (Johnston's organ) is located in the second segment of the antenna (A2) and consists of functional units called scolopidia. These are attached to the cuticle of the second antennal segment and to the A2/A3 joint. Sound particles cause the displacement of the antennal arista, which induces the third antennal segment to rotate (arrow). This acoustic, stimulus-induced rotation applies force to the cilia of mechanosensory neurons in the scolopidia, resulting in the firing of action potentials along the antennal nerve. A single scolopidium is highlighted in the light green box. Each scolopidium is attached to the antenna cuticle (arrows) by a cap cell (red) and a ligament cell (gray), as detailed in C. (C) Cellular components of an individual scolopium. Two to three mechanosensory neurons (two neurons are shown) have their ciliary dendrites enclosed by an actin-rich scolopale cell. Mechanosensory neurons express several ion channels that regulate mechanosensation, including Nan/Iav (green) and NompC (yellow). Scolopale cells form septate junctions with the ligament and cap cells at the basal and apical ends of the scolopidium, respectively, ensuring the formation of an enclosed scolopale space between the scolopale cell and neuronal cilia. The ciliary dendrites of each neuron insert into an acellular cap that contains the NompA glycoprotein (red), which is anchored to the A2/A3 joint by a cap cell. The scolopidium is attached to the A2 cuticle at its proximal end by a ligament cell. Approximately 200 scolopidia are located in Johnston's organ.

Johnston's organ, the largest chordotonal organ (Box 1) in *Drosophila*, is located in the second antennal segment (Box 1) and consists of ∼200 functional units or scolopidia (Kernan, 2007). Each scolopidium is suspended between the cuticle of the second antennal segment and the insertion of the third antennal segment (Fig. 1A,B) (Todi et al., 2004). During courtship, male flies beat their wings, causing the air to move, which in turn causes the feather-like arista (Box 1) on the third antennal segment of the female to vibrate. The resulting movement of the third antennal segment applies force to the scolopidia as the a2/a3 joint rotates (Boekhoff-Falk and Eberl, 2014; Eberl and Boekhoff-Falk, 2007). This stretch is transmitted to the mechanosensory cilia of sensory neurons, opening stretch-gated mechanotransduction channels (Kernan et al., 1994; Kernan, 2007). A transient receptor potential (TRP) channel – TRPN1 (also known as NompC) – was first identified as a candidate component of a mechanosensitive channel in a genetic screen in *Drosophila* (Walker et al., 2000), and was subsequently shown to be expressed in the distal region of the ciliary dendrite of Johnston's organ neurons (Lee et al., 2010). Mutation of *Drosophila nompC* attenuates, but does not completely abolish, sound-evoked potentials (Eberl et al., 2000; Effertz et al., 2012, 2011; Göpfert et al., 2006; Lee et al., 2010; Liang et al., 2011). Two TRPV family members, Nanchung (Nan) and Inactive (Iav) are also expressed in Johnston's organ neurons, although in a more proximal region of the ciliated dendrite than NompC (Cheng et al., 2010; Gong et al., 2004; Lee et al., 2010; Liang et al., 2011). Nan and Iav are also required for the generation of sound-evoked potentials in the antennal nerve (Gong et al., 2004; Kim, 2007; Kim et al., 2003). It is less clear whether Nan and Iav can be directly gated by mechanical force, as studies in which either or both channels were expressed in heterologous cells reached different conclusions over the degree to which the channels could be opened by osmotic stress [compare Gong et al. (2004) and Kim et al., (2003) with Nesterov et al. (2015)]. The physical separation of the TRPN and TRPV channels in the dendrite of Johnston's organ neurons, together with the effects of these mutations on sound-evoked potentials and on active amplification, has led to the idea that NompC plays a direct role in mechanotransduction (Effertz et al., 2012; Göpfert et al., 2006), whereas Nan and Iav, which can form heteromers (Gong et al., 2004), are thought to amplify weak signals transmitted from the NompC complex (Gong et al., 2004; Göpfert et al., 2006; Kamikouchi et al., 2009; Kim et al., 2003).

Despite the progress made in characterizing the properties of these channels, it has been hard to resolve the relative contributions of these three channels to mechanotransduction, in part because of the technical challenges involved in recording from single auditory neurons in Johnston's organ. A recent innovation by Wilson and colleagues (Lehnert et al., 2013) exploited the fact that auditory neurons terminate on, and are dye-coupled to, a single giant fiber neuron in the brain (Kamikouchi et al., 2009; Tootoonian et al., 2012). Recording the activity of this neuron allows currents from Johnston's organ neurons to be indirectly measured. Results from this approach suggest that Nan and Iav are required to respond to sound, whereas NompC is required for the modulation and amplification of force generated during sound reception (Lehnert et al., 2013). It is notable that NompC contains numerous ankyrin repeats (Cheng et al., 2010; Liang et al., 2013; Zhang et al., 2015) that might interact with motile components of the ciliated dendrite to modulate force, an idea that is supported circumstantially by the presence of NompC in the distal cilium, between the site of cilia insertion, and by more proximal location of the Nan/Iav complex in the cilium.

**Fig. 2.**
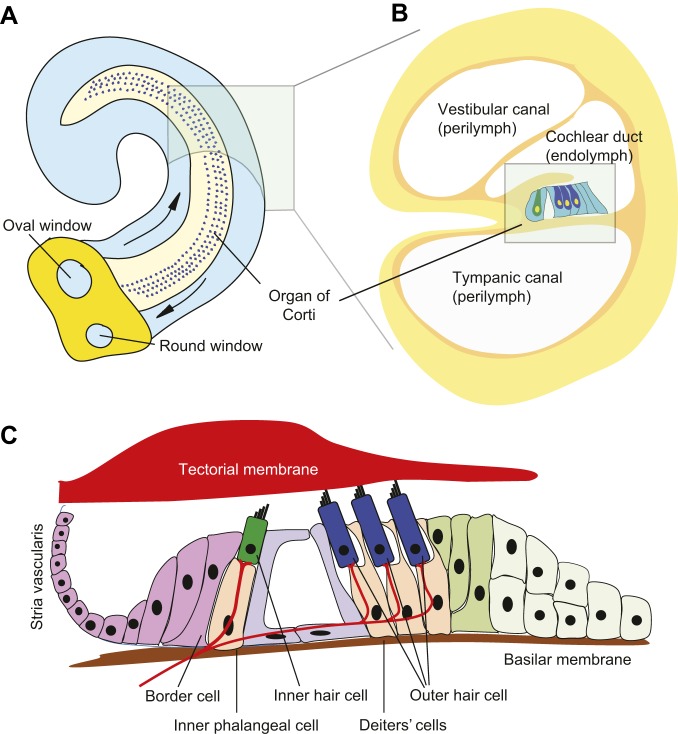
**Structure of the mammalian auditory organ.** (A) In the mouse inner ear, the organ of Corti is located on the basilar membrane, which runs the length of the cochlear duct. Mechanical waves conducted by the middle ear bones are transmitted to the oval window and travel through the perilymph surrounding the cochlear duct (arrows). Compression of the oval window is matched by a corresponding outward movement at the round window. (B) A cross-section of the cochlear duct, showing three rows of outer hair cells (dark blue) and one row of inner hair cells (dark green). (C) A magnified view of the arrangement of hair cells in the organ of Corti. Inner hair cells are surrounded by inner phalangeal and border cells and are separated from the outer hair cells by two pillar cells that together form the tunnel of Corti. Outer hair cells are surrounded by Deiters' cells. Inner and outer hair cells receive afferent and efferent innervation, respectively. Stereocilia located on the apical surface of hair cells are embedded into the tectorial membrane (outer hair cells) or lie just beneath the membrane (inner hair cells). During sound reception, the sound-induced vibration of the basilar membrane applies mechanical force to the tip links of the stereocilia, causing an influx of potassium and calcium and the depolarization of hair cells. The potassium gradient in the cochlear duct endolymph is maintained by the stria vascularis in the lateral wall of the cochlear duct.

Unlike the *Drosophila* hearing organ, which responds optimally to a narrow frequency range of the beating male wing, the mammalian inner ear has evolved to respond to a wide range of sound frequencies. The hearing organ of the cochlea, the organ of Corti, sits on a thin basilar membrane that runs the length of the cochlear duct (Fig. 1B). Both the thickness and width of the basilar membrane change along the length of the cochlear duct (Dallos, 1996); therefore, it can resonate in response to many different frequencies, allowing the basilar membrane to transform complex sounds into their component sound frequencies (Reichenbach and Hudspeth, 2014). The rising and falling oscillation of the basilar membrane in response to mechanical waves traveling along the cochlea is transmitted to the mechanosensory hair cells of the organ of Corti (Fettiplace and Hackney, 2006). One row of inner hair cells runs the length of the organ of Corti, and these cells form synapses with sensory afferent neurons of the acoustic (spiral) ganglion (Goodrich, 2016), while three rows of outer hair cells receive mostly efferent input from the brainstem (Basch et al., 2016; Yu and Goodrich, 2014). Hair cells bear elongated microvilli or stereocilia on their apical surface that insert into an acellular tectorial membrane above them (Fig. 2). As the basilar membrane vibrates, the motion of the organ of Corti causes the stereociliary bundles of the hair cells to be displaced by contact with the tectorial membrane (outer hair cells) or fluid flow (inner hair cells). This opens mechanically gated ion channels in the tips of the stereocilia (Corey and Holt, 2016; Fettiplace, 2016; Wu and Muller, 2016). Adjacent stereocilia connect to each other close to their tips by a tip link protein complex consisting of protocadherin 15 and cadherin 23 (Pepermans and Petit, 2015). This complex allows the opening of mechanotransduction channels to be coordinated as the hair bundle is displaced (Hudspeth, 1997, 2008). As in *Drosophila*, the identity of the mechanosensitive ion channels in these hair cells is still being investigated and debated (Corey and Holt, 2016; Fettiplace, 2016; Wu and Muller, 2016). Current work is focused on four transmembrane proteins, transmembrane inner ear (TMIE), tetraspan membrane protein of hair cell stereocilia/lipoma HMGIC fusion partner-like 5 (TMHS; Lhfpl5) and transmembrane channel-like 1 and 2 (TMC1/2), all of which are necessary for hearing in mice (Kawashima et al., 2011; Kurima et al., 2015; Xiong et al., 2012; Zhao et al., 2014). It is possible that other proteins, such as the large transmembrane protein Piezo2, might also contribute to mechanotransduction in other parts of the hair cell (Wu et al., 2017).

Although the appearance and organization of mammalian and *Drosophila* hearing organs are clearly very different, they share a number of genes that regulate their formation and cellular functions. We describe some of these in the following section.

## Cellular and molecular similarities between *Drosophila* and mammalian hearing organs

Despite being separated by several hundred million years of evolutionary time, the mechanosensitive cells and supporting cells of mammalian and *Drosophila* hearing organs are specified by similar sets of transcription factors, and have similarities in the accessory cells and proteins that allow these receptor cells to be mechanically gated. We first compare the axonless hair cells of vertebrates with the mechanosensitive neurons of *Drosophila* and then describe similarities in how these cells are formed and how they function.

Vertebrate hair cells are secondary receptor cells: they elaborate neither axons nor dendrites but are innervated by axons of bipolar sensory neurons that also send processes to brainstem nuclei. In addition to their apical stereociliary bundle, they also have a true cilium, the kinocilium, which disappears in some hair cell types as they mature, including inner and outer hair cells of the mammalian cochlea (Nayak et al., 2007). Among invertebrates, only insects and crustaceans have true hearing; however, other invertebrate groups possess mechanoreceptor cells with similar apical specializations that can detect gravity and acceleration. These receptor cells are also sometimes referred to as hair cells, although their homology to true vertebrate hair cells is still under debate (Burighel et al., 2011, 2008, 2003; Fritzsch and Straka, 2014; Manley and Ladher, 2007). These invertebrate ‘hair cells’ can be either secondary receptors or primary sensory neurons that have intrinsic mechanosensitive specializations (Burighel et al., 2011). These specializations can be actin-rich structures similar to vertebrate stereocilia or mechanosensitive organelles made up of one or more true cilia, reminiscent of vertebrate photoreceptors. Some nonvertebrate groups, such as urochordates and cephalopod mollusks, use both kinds of receptor cells in their sensory organs (Budelmann and Thies, 1977; Burighel et al., 2011). In contrast, secondary receptor cells have not been observed in insects and crustaceans (Fritzsch and Straka, 2014), and these groups instead have a wide range of mechanoreceptive sensory neurons arranged in clusters all over their bodies (Kernan, 2007), including the ciliated sensory neurons in scolopidia of Johnston's organ. It is not clear whether a mechanosensitive sensory neuron represents the ancestral cell type from which hair cells and sensory neurons emerged as sister cell types in multiple taxa, or whether secondary mechanosensitive cells were lost in the arthropod lineage (Arendt et al., 2016).

Despite the structural differences between insect and vertebrate sound-detecting cells, molecular evidence suggests that certain aspects of the development of the various cell types found in auditory organs are conserved between invertebrates and vertebrates (Bechstedt and Howard, 2008; Bokolia and Mishra, 2015; Caldwell and Eberl, 2002; Lewis and Steel, 2012) (Table 1). The *atonal* (*ato*) basic helix-loop-helix (bHLH) transcription factor was discovered in *Drosophila* as a proneural gene required for the formation of photoreceptors and of some mechanoreceptors (Jarman et al., 1993, 1994). In *Drosophila*, the loss of *ato* leads to loss of all chordotonal organs, including Johnston's organ, while its misexpression results in the formation of ectopic chordotonal organs (Jarman et al., 1993). Similarly, the vertebrate homolog of *ato*, *Atoh1*, is both necessary and sufficient for the differentiation and survival of hair cells (Bermingham et al., 1999; Cai et al., 2013). Remarkably, *Drosophila* *ato* and mouse *Atoh1* can functionally replace each other in loss-of-function mutants (Ben-Arie et al., 2000; Wang et al., 2002), and are both regulated through conserved post-translational regulation (Quan et al., 2016), even though they share little homology outside the bHLH domain. A conserved post-translational control of the temporal dynamics of *ato/Atoh1* expression during neurogenesis further supports the functional similarities between *ato* and *Atoh1* (Quan et al., 2016). The conservation between *Drosophila* *ato* and the vertebrate *Atoh1* gene regulatory networks is reflected by conservation of some of their downstream target genes. The zinc finger transcription factor *senseless* (*sens*)/*Gfi1*, which can be regulated by *ato/Atoh1*, is required for the development of the chordotonal organ in *Drosophila* embryos and for the survival of hair cells in the mouse (Acar et al., 2006; Jafar-Nejad et al., 2003, 2006; Nolo et al., 2000; Wallis et al., 2003; Wang et al., 2002). Regulatory factor X (Rfx) is another downstream transcription factor activated by Atonal (Jarman and Groves, 2013; Laurençon et al., 2007) that regulates many ciliogenic genes in a variety of organisms, including in *Drosophila* sensory neurons (Dubruille et al., 2002; Vandaele et al., 2001). A recent study showed that combined loss of *Rfx1* and *Rfx3* in mice leads to a rapid loss of initially well-formed outer hair cells in the mouse cochlea and causes deafness (Elkon et al., 2015).

**Table 1.**
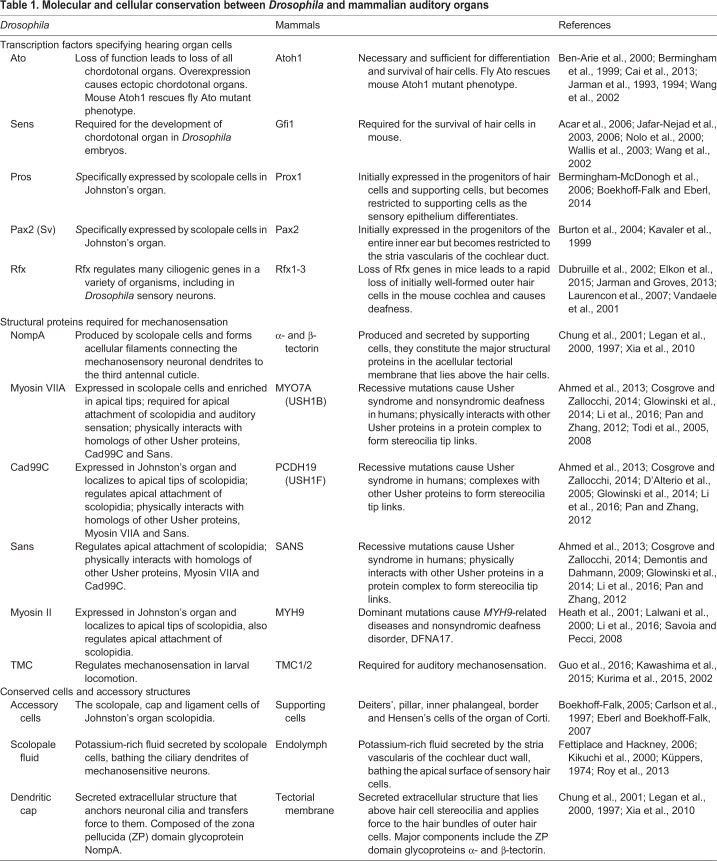
**Molecular and cellular conservation between *Drosophila* and mammalian auditory organs**

The division of labor between mechanosensitive secondary receptor cells and their afferent sensory neurons in mammals and some invertebrate taxa is reflected by an analogous division of labor between the transcription factors that specify the neuronal and sensory lineages. *Neurog1* is a bHLH factor closely related to *Atoh1* and is expressed in the progenitor cells of the inner ear primordium shortly before they delaminate as neuroblasts (Zheng and Gao, 2000). Its expression is then extinguished and replaced by another closely related bHLH gene, *Neurod1*; both genes are necessary for the generation of inner ear neurons (Kim et al., 2001; Liu et al., 2000). The Atoh, Neurogenin and NeuroD bHLH transcription factor families are ancient, with members of each family observed in basal taxa, such as sponges (Simionato et al., 2008, 2007). Arthropods appear to have dispensed with the need for the Neurog genes in sensory development; the one identified member of this family in *Drosophila*, *target of pox neuro* (*tap*), appears to function in neurite outgrowth rather than in neuronal specification (Gautier et al., 1997; Yuan et al., 2016). In contrast, *Drosophila ato* has undergone duplication to give rise to two additional orthologs: *absent multidendritic and olfactory sensilla* (*amos*) and *cousin of atonal* (*cato*) (Jarman and Groves, 2013). Although the Atoh, Neurogenin and NeuroD bHLH families are more closely related to each other than to other bHLH genes, they nevertheless have extremely specific functions. For example, while *Drosophila ato* can functionally replace *Atoh1* in mice, and vice versa (Ben-Arie et al., 2000; Wang et al., 2002), *Neurog1* can only rescue loss of *Atoh1* in mice to a very modest degree (Jahan et al., 2012, 2015), suggesting that Neurog1 is only able to activate a subset of Atoh1 target genes. Thus, these different bHLH factors can specify different cell fates – neuron or hair cell – depending on the binding affinities and range of their targets.

A common feature of sensory systems is the presence of accessory cells that surround, support, nourish or modulate the output of sensory cells. In each scolopidium of Johnston's organ, neuronal cilia are enclosed by a tube-like scolopale cell (Fig. 1). The shape of the scolopale cell is thought to be maintained by a cage-like scolopale consisting of actin rods, microtubules and some microtubule-associated proteins (Todi et al., 2004; Wolfrum, 1997). Scolopale cells form septate junctions with the ligament and cap cells at the basal and apical ends of the scolopidium, respectively, ensuring the formation of an enclosed scolopale space between the scolopale cell and neuronal cilia (Boekhoff-Falk, 2005; Carlson et al., 1997; Eberl and Boekhoff-Falk, 2007; Todi et al., 2004). Although the ionic composition of the scolopale space has not been measured directly in *Drosophila*, studies of the fluid that bathes a variety of insect and crustacean mechanoreceptors have reported that this fluid has a positive potential of about +70 mV relative to the hemolymph (Thurm and Wessel, 1979). In addition, studies of blowfly mechanoreceptor organs have found such fluid to be high in potassium (Küppers, 1974), the concentration of which is actively maintained by transport ATPases (French, 1988; Thurm, 1974; Wieczorek, 1982). Scolopale cells express Na^+^/K^+^ ATPases that localize to their ablumenal plasma membrane and are required for hearing in *Drosophila*. These Na^+^ pumps function to actively transport K^+^ into the scolopale space to maintain ion homeostasis (Roy et al., 2013). In the mammalian organ of Corti, inner hair cells and outer hair cells are surrounded by different types of supporting cells: inner phalangeal and border cells surround the inner hair cells, while Deiters' cells surround the outer hair cells (Fig. 2), and inner and outer pillar cells located between these two domains form the tunnel of Corti. During sound perception, sound-induced vibration of the basilar membrane applies force to the tip links of the stereocilia. This results in their deflection, in the opening of cation channels in the stereocilia and in the influx of K^+^ and Ca^2+^ from the surrounding potassium-enriched endolymph (Fettiplace and Hackney, 2006). Potassium homeostasis in the endolymph is crucial for the maintenance of the endocochlear potential and for hair cell function. The K^+^ that enters hair cells is recycled through the supporting cells and is ultimately transported back into the endolymph by cells in the stria vascularis of the lateral wall of the cochlear duct (Kikuchi et al., 2000). It is not clear whether the K^+^-rich extracellular fluid of the endolymph and scolopale space reflect a common or convergent evolutionary origin. For example, sensory hair cells of the lateral line organs of vertebrates are not enclosed, but open to the external fluid environment, and a number of studies measuring the endolymph-like fluid bathing cephalopod statocyst sensory organs have found it to have low levels of K^+^ compared with Na^+^ (reviewed in Vinnikov, 2011).

Some molecular and cellular similarities have been identified between scolopale cells and cochlear-supporting cells. *prospero* (*pros*), a homeodomain transcription factor, is specifically expressed by scolopale cells in Johnston's organ (Boekhoff-Falk and Eberl, 2014). The mammalian homolog of *pros*, *Prox1*, is initially expressed in the progenitors of hair cells and supporting cells, but becomes restricted to supporting cells as the sensory epithelium differentiates (Bermingham-McDonogh et al., 2006). On the apical side of Johnston's organ, cap cells participate in the coupling of neuronal cilia to the cuticle of the third antennal segment, and help impart mechanical force to the cilia during sound perception. *nompA* encodes a zona pellucida (ZP) domain glycoprotein that is produced and secreted by scolopale cells. It forms acellular filaments that connect the mechanosensory neuronal dendrites to the third antennal cuticle (Chung et al., 2001). Loss of NompA leads to the detachment of both the scolopidia from the third antennal segment cuticle and of the ciliary dendrites from the cuticular structures in other sensory organs, such as the external sensory organs and other campaniform organs (Chung et al., 2001). Mammals also have ZP domain-containing proteins – α- and β-tectorin (Legan et al., 1997) – which are produced and secreted by supporting cells, and are the major structural proteins in the acellular tectorial membrane that lies above the hair cells, and that comes into contact with the hair bundles of the outer hair cells. Loss of α-tectorin in mice leads to the detachment of hair cells from the tectorial membranes (Legan et al., 2000; Xia et al., 2010). Intriguingly, a *Caenorhabditis* *elegans* ZP domain protein, DYF-7, resembles tectorins and anchors the tips of sensory dendrites to the body wall as the neuronal cell bodies in the developing amphid migrate away (Heiman and Shaham, 2009). In addition, the transmembrane channel-like (TMC) proteins are required for auditory mechanosensation in mice and humans (Kawashima et al., 2015). Although it has yet to be shown that the *Drosophila* homolog of the TMC proteins functions directly in auditory mechanosensation, it has been shown to regulate mechanosensation in larval locomotion (Guo et al., 2016).

A striking illustration of the molecular and genetic similarities between *Drosophila* and mammalian hearing organs comes from the work of Göpfert and colleagues (Senthilan et al., 2012). Here, a microarray survey of genes expressed in Johnston's organ of wild-type and *ato* mutant flies identified 274 genes expressed in this auditory organ. Approximately 20% of these genes had a human ortholog implicated in hearing loss. This suggests that the fruit fly might be a rich resource for identifying and characterizing genes involved in human deafness. In the next section, we describe recent work in which forward genetic screens identified a *Drosophila* gene that linked together two myosin genes previously implicated in human syndromic hearing loss.

## Can *Drosophila* be used to model hereditary deafness and hearing loss?

The molecular and functional similarities between Johnston's organ and the mammalian cochlea summarized above suggest that some genes implicated in human deafness might have similar functions in Johnston's organ. Here, we focus on two particular forms of syndromic hearing loss in humans, Usher syndrome and *MYH9*-related disorders, as these well-characterized syndromes provide evidence of such functional similarity and highlight the potential advantages of using *Drosophila* as a model for studying hereditary deafness.

## Conservation of Usher syndrome proteins and their interactions in *Drosophila*

Usher syndrome is an autosomal recessive genetic disease [Online Mendelian Inheritance in Man (OMIM) #276900, #276904, #601067, #276901, #605472, #611383] that is characterized by varying degrees of deafness and retinitis pigmentosa-induced vision loss, and is the leading cause of deaf-blindness in humans (Boughman et al., 1983; Hartong et al., 2006; Keats and Corey, 1999; Kimberling et al., 2010). Based on the severity of clinical symptoms in patients, Usher syndrome is subdivided into three types: USH1, 2 and 3. To date, 16 genetic loci have been associated with this syndrome: nine for USH1, three for USH2, two for USH3 and two that have yet to be identified (Cosgrove and Zallocchi, 2014; Mathur and Yang, 2015). Some missense mutations of Usher (USH) genes that retain residual function are also associated with nonsyndromic forms of deafness that do not cause vision loss (see, for example, Schultz et al., 2011).

**Fig. 3.**
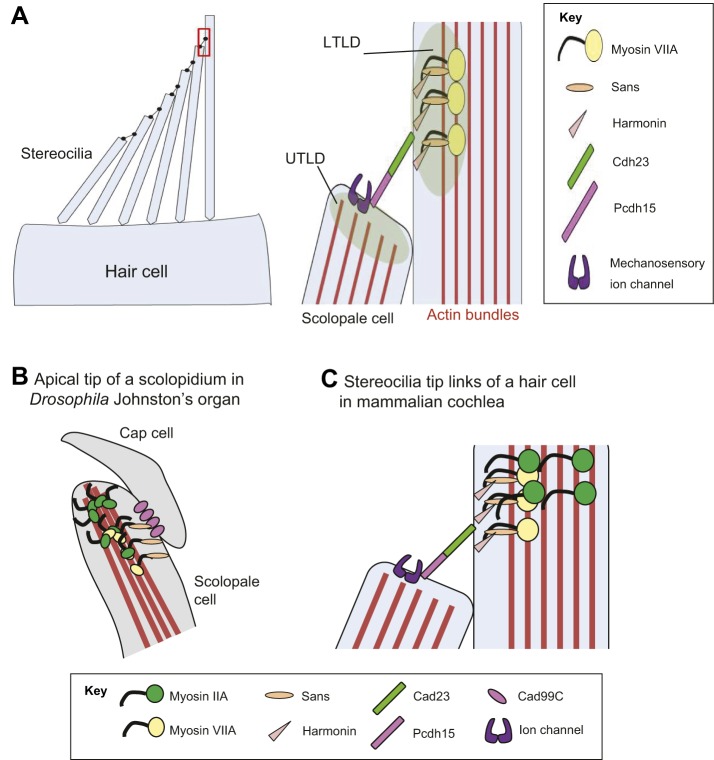
**USH1 proteins and Myosin II function in the apical region of *Drosophila* scolopidia and mammalian stereocilia.** (A) Left: a schematic of the apical surface of a hair bundle in a single mouse hair cell, showing individual stereocilia. The red box outlines the tip link region, which is expanded to high magnification on the right. Right: USH proteins form a protein complex and link the upper tip link density (UTLD) in the taller stereocilia to the lower tip link density (LTLD) of the neighboring shorter stereocilia in the same hair cell. In the prevailing model of USH protein interactions, the Cdh23-Pchd15 heterodimer is located directly or in close proximity to other protein components of the mechanotransduction (MET) channel complex, including TMIE and TMHS. The adaptor proteins harmonin and Sans link Cdh23 to myosin VIIA and hence to the actin core of the stereocilium. (B) A schematic of a scolopidium. In *Drosophila*, Myosin VIIA and Myosin II are present in the apical regions of the scolopidia of Johnston's organ and are enriched at the tips of scolopale cells, where they contact the cap cell. Myosin II ubiquitination promotes its interaction with Myosin VIIA, the levels of which are crucial for anchoring the apical junction complexes of the scolopidia. The motor activity of these myosins might also be required to transport the myosin complex to the tips of the scolopale cell. Both Myosin VIIA and Myosin II likely bind to actin bundles in the scolopale cells and regulate the apical attachment of scolopidia. Two *Drosophila* homologs of Usher syndrome type I proteins, Cad99C (Pcdh15) and Sans, interact with Myosin VIIA in a protein complex. It is not clear whether Cad99C mediates attachment to neuronal cilia or cap cells as a homodimer or as a heterodimer with another adhesion molecule. (C) In mammalian hair cells, a USH1 protein complex that includes myosin VIIA, Sans, harmonin and Cdh23 is located close to the stereocilia tips. By analogy with *Drosophila*, we propose that myosin IIA interacts with myosin VIIA, and that this interaction is promoted by myosin II ubiquitination. The motor activity of either myosin might be required for the transport of the myosin VIIA-myosin II-USH1 protein complex to the stereocilia tips.

USH proteins regulate a variety of cellular processes, including actin-based trafficking, cell adhesion, scaffolding, and G-protein- or Ca^2+^-mediated signaling (Table 2). Evidence from colocalization data, *in vitro* interaction studies, and mouse genetic studies suggest that USH proteins interact with each other to form multiprotein complexes in a variety of cell types (Fig. 3A) (Ahmed et al., 2013; Pan and Zhang, 2012). In mature mammalian hair cells, many Usher syndrome proteins localize to the tips of stereocilia where mechanotransduction takes place (Fig. 3A). Adjacent stereocilia are connected by complexes at their tips, called tip links, which are composed of cadherin 23 (USH1D; Cdh23) and protocadherin 15 (USH1F; Pcdh15). Protocadherin 15 is thought to interact with components of the mechanotransduction channel (TMHS), and both ends of the tip links are indirectly coupled to the actin core of the stereocilia through other Usher proteins, including myosin VIIA (USH1B; Myo7A), Sans (USH1G), harmonin (USH1C), Cib2 and whirlin (USH2D) (Ahmed et al., 2013; Cosgrove and Zallocchi, 2014).

**Table 2.**
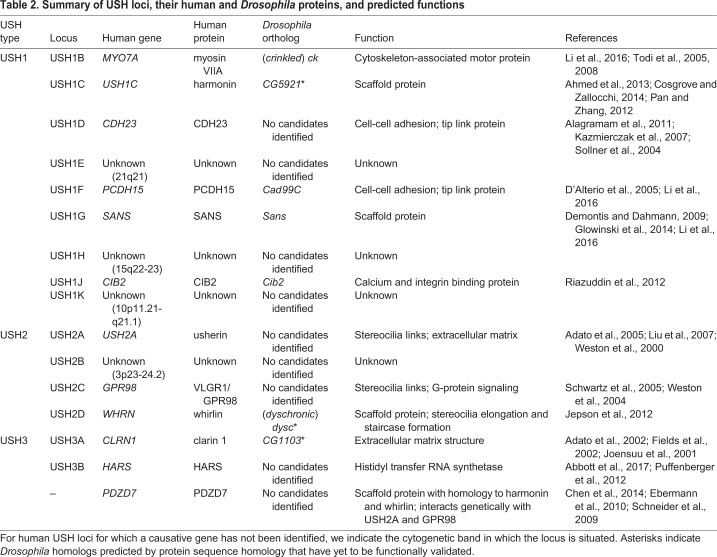
**Summary of USH loci, their human and *Drosophila* proteins, and predicted functions**

**Fig. 4.**
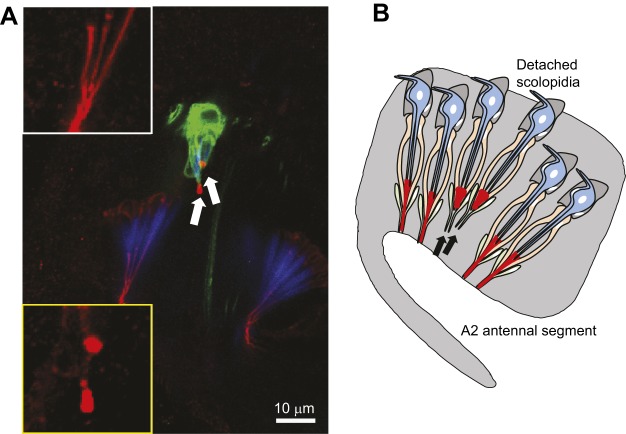
**Apical detachment of scolopidia in *DrosophilaUbr3* mutants.** (A) A confocal image of the second antennal segment of a mosaic adult *Drosophila* in which GFP^+^ marked cells are *Ubr3^−/−^* (mutant) and GFP^–^ cells are *Ubr3^+/−^* (wild-type control). The apical junction protein NompA is shown in red and actin (scolopale cells) in blue. Arrows indicate two detached *Ubr3^−/−^* scolopidia, which also exhibit abnormal puncta pattern of NompA. The white outline box (upper) and yellow outline box (lower) show high-power images of the NompA pattern in the apical junction from wild-type or *Ubr3* mutant scolopidia, respectively. (B) A schematic summary of the apical detachment of scolopidia in *Ubr3* and other Usher mutants, such as *myosin VIIA* and *Cad99c*. Arrows indicate detached scolopidia. Image in A taken from Li et al. (2016).

The *Drosophila* genome harbors homologs of several Usher genes and some of these have been characterized in Johnston's organ (Table 2). The *Drosophila* homolog of myosin VIIA [*myosin VIIA*; also known as *crinkled* (*ck*)] is required for hearing: loss of *ck* abolishes a fly's auditory responses and causes the scolopidia to detach from the cuticle of the a2/a3 antennal joint in Johnston's organ (Li et al., 2016; Todi et al., 2005, 2008) (Fig. 4). Interestingly, Myosin VIIA is predominantly expressed in the actin-rich scolopale cells and is enriched at their apical tips close to where a scolopidium inserts into the cuticle (Li et al., 2016), a pattern that is superficially similar to the localization of myosin VIIA in the actin-rich stereocilia tips of mammalian hair cells. However, whereas myosin VIIA functions in hair cell stereocilia to mechanically couple mechanotransduction channels to the actin cytoskeleton, Myosin VIIA is unlikely to have the same function in scolopale cells, which ensheath the mechanosensitive cilia of the Johnston's organ sensory neurons and secrete the dendritic cap that anchors them to the cap cell and cuticle. However, it is likely that myosin VIIA serves an anchoring role in both cell types, because it is a high duty ratio motor – i.e. it predominantly binds to actin filaments rather than processing along them (Haithcock et al., 2011; Watanabe et al., 2006; Yang et al., 2006). Pcdh15 and Sans also have *Drosophila* homologs, named *Cad99C* and *Sans*, respectively (D'Alterio et al., 2005; Demontis and Dahmann, 2009). Recent studies have shown that Cad99C, Sans and Myosin VIIA form a complex in Johnston's organ and in the ovary of *Drosophila* (Glowinski et al., 2014; Li et al., 2016). In Johnston's organ, a subtle, low-penetrant phenotype of scolopidial detachment can be observed in *Cad99C* mutants, resembling that of *myosin VIIA* mutants. Cad99C localizes to the apical tips of scolopidia, reminiscent of the localization of Pcdh15 to the tip link region in mammalian hair cells. In addition, *myosin VIIA*, *Cad99C* and *Sans* genetically interact with *Ubr3*, a regulator of Usher proteins in Johnston's organ (Li et al., 2016).

These data suggest that USH1 proteins are not only conserved in *Drosophila*, but that their interactions are important for the development and function of the *Drosophila* auditory apparatus. USH1 proteins appear to interact in the scolopidia tips to link the scolopale cell and cap cell, which might stabilize the scolopidial unit during mechanotransduction where force is applied to the neuronal cilia through the extracellular dendritic cap (Fig. 3B,C). This function of USH proteins in Johnston's organ – where the proteins appear to mediate intercellular interaction and attachment – is somewhat different to their functions in the mammalian hair cell, in which the USH complex acts intracellularly to anchor adjacent stereocilia.

Several other USH genes have *Drosophila* homologs, such as harmonin (*CG5921*), *Cib2* (*cib2*), whirlin (*dyschronic*) and clarin 1 (*CG1103*) (Table 2). Although *cib2* is expressed in the eye and is required for normal phototransduction in *Drosophila*, whether these homologs are also present in the scolopidia of Johnston's organ and/or participate in hearing remains unclear. Other vertebrate USH proteins that are present in the ankle links that connect the base of stereocilia, such as usherin and VLGR1/GPR98 (Adgrv1), do not appear to have any obvious homologs in *Drosophila* (Table 2). Moreover, the mechanism by which *Drosophila* USH proteins cause scolopidial attachment – for example, how the scolopidium inserts into the cuticle, or whether additional cadherins act with Cad99C/Pcdh15 – remains unclear. Nevertheless, the clear and quantifiable phenotypes seen in Usher syndrome gene mutants in *Drosophila* (Fig. 4), and the strong genetic interactions between Usher genes, supports the use of *Drosophila*, and the Johnston's organ, as a model in which to study the functions of known and newly identified Usher genes and to identify their regulators. Finally, one recently reported unexpected finding is that USH homologs in the *Drosophila* hearing organ genetically and physically interact with Myosin II (Li et al., 2016), the mammalian homologs of which also play a role in hearing. We describe the function of Myosin II in hearing in flies and mammals in the next section.

## *MYH9*-related disease

Autosomal dominant *MYH9*-related diseases are caused by mutations in *MYH9*, which encodes nonmuscle myosin IIA. These diseases are characterized by large platelets and thrombocytopenia from birth, and are associated with a variable onset of progressive sensorineural hearing loss, presenile cataracts, elevated liver enzymes and renal disease, which initially manifests as a glomerular nephropathy (Heath et al., 2001). Prior to the identification of the underlying molecular cause, individuals with *MYH9*-related disease were variously diagnosed as having Epstein syndrome, Fechtner syndrome, May-Hegglin anomaly or Sebastian syndrome (Heath et al., 2001; Newell-Litwa et al., 2015). The onset of hearing loss in patients with a *MYH9*-related disease can occur over a wide age range, from the first to the sixth decade (Savoia and Pecci, 2008), and frequently progresses over time. In addition, a dominant mutation, *MYH9^R705H^*, causes the nonsyndromic deafness disorder called DFNA17 (Lalwani et al., 2000).

Mammals have three isoforms of nonmuscle myosin II encoded by three different genes: NMIIA (encoded by *MYH9*), NMIIB (encoded by *MYH10*) and NMIIC (encoded by *MYH14*). Mice that are homozygous for a *Myh9* null mutation die during embryogenesis by embryonic day (E) 7.5 (Conti et al., 2004; Mhatre et al., 2007). Mice heterozygous for *Myh9* do not exhibit any hearing loss or changes in age-dependent hearing thresholds (Mhatre et al., 2007). This suggests that *MYH9*-related diseases are not caused by *MYH9* haplo-insufficiency. However, knock-in mice, in which one *Myh9* allele has been modified to contain a specific dominant pathogenic *Myh9* mutation, R702C, develop defects, including hearing loss and kidney disease, that are similar to symptoms associated with several *MYH9*-related syndromes such as May-Hegglin anomaly (Suzuki et al., 2013). Although *Myh9* is expressed in hair cells and NMIIA localizes to the stereocilia and apical surface of the hair cell (Li et al., 2016; Meyer Zum Gottesberge and Hansen, 2014; Mhatre et al., 2006), NMIIA is also broadly expressed in many other parts of the cochlea. It is therefore possible that the deafness that occurs in *MYH9*-related diseases might not be directly caused by hair cell defects. Thus, the molecular mechanism by which *MYH9* mutations cause hearing loss remains unclear.

The single *Drosophila* homolog of NMIIA is encoded by the *myosin II* gene, also known as *zipper*. This gene is expressed in Johnston's organ, and Myosin II is enriched at the apical tips of scolopidia, adjacent to Myosin VIIA (Fig. 3B) (Li et al., 2016). Myosin II is also required for the normal apical attachment of scolopidia. Consistent with this, the gene encoding the regulatory light chain of Myosin II, *spaghetti squash* (*sqh*), genetically interacts with *myosin VIIA* in Johnston's organ and affects hearing (Todi et al., 2008). Recent studies in *Drosophila* show that Myosin II physically interacts with Myosin VIIA, probably through the mono-ubiquitination of Myosin II, which is regulated by the E3 ligase, Ubr3 (Li et al., 2016). The myosin IIA-myosin VIIA interaction and the mono-ubiquitination of myosin IIA also occur in the mammalian cochlea (Li et al., 2016), although an interaction between these two myosins in stereocilia (Fig. 3C) remains speculative. The overexpression of different pathogenic variants of Myosin II in Johnston's organ leads to the apical detachment of scolopidia, which also occurs in *Drosophila* USH mutants (Li et al., 2016). These data indicate that Myosin II functions in *Drosophila* hearing, together with Myosin VIIA and/or other USH1 proteins; this interaction might also underlie the pathogenic effects of some pathogenic variants of myosin IIA in *MYH9*-related disease. The clear cut and quantitative scolopidial detachment phenotype (Fig. 4) associated with *myosin II* and *myosin VIIA* mutations in Johnston's organ provides a quick and easy assay with which to investigate the molecular function of Myosin II in hearing and the mechanisms by which pathogenic Myosin II variants cause hearing loss. In the next section, we describe new technology that exploits the *Drosophila* to human conservation of genes implicated in hearing loss to rapidly test the function of human genes and their variants in *Drosophila.*

## New approaches for modeling human deafness in *Drosophila*

**Fig. 5.**
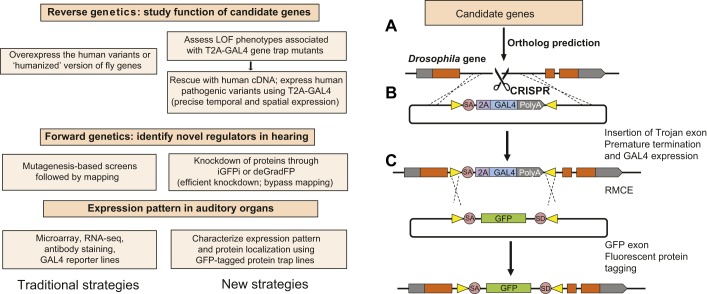
**Approaches to functionally test candidate mammalian deafness genes in *Drosophila*.** (A) Candidate genes can include known deafness genes or mouse genes with deafness phenotypes. Their *Drosophila* homologs are predicted by protein sequence homology. (B) An artificial exon that acts as a gene trap can be inserted into a MiMIC line by recombination-mediated cassette exchange (RMCE; Venken et al., 2011) or by CRISPR-mediated homology-directed repair, which prematurely terminates transcription of the endogenous gene through a splice acceptor site (SA) and poly-adenylation signal (PolyA), and expresses the GAL4 transcriptional activator as part of the endogenous transcript through a picornavirus self-cleaving 2A peptide sequence (2A). (C) Gene trap/GAL4 lines are screened for hearing phenotypes. The GAL4 protein expressed by the endogenous regulatory elements of the gene of interest can also be used to drive the expression of transgenes under the control of the UAS DNA element inserted elsewhere in the fly genome. These transgenes can be wild type, or pathogenic human or mouse orthologs, and the insertion of GAL4 into the endogenous locus allows the transgene to be expressed in a spatiotemporal pattern that is identical to the endogenous *Drosophila* gene. RMCE through attP and attB sites (yellow triangles) can convert the Trojan exon into an exon containing GFP flanked by SA and splice donor (SD) sites, to reveal the cellular and subcellular localization of the trapped gene (Nagarkar-Jaiswal et al., 2015b). The GFP exon can also be used to knockdown the encoded transcript or protein by *in vivo* GFP interference (iGFPi) or the deGradFP system, respectively. LOF, loss of function; RNA-seq, RNA sequencing.

Mutagenesis-based screens in mice and *Drosophila* have identified candidate genes responsible for hearing loss and age-related hearing loss (Eberl et al., 1997; Hrabe de Angelis et al., 2000; Kernan et al., 1994; Nolan et al., 2000; Potter et al., 2016; Senthilan et al., 2012). Researchers used a large-scale lethal mosaic screen (Box 1) to isolate mutations that affect the *Drosophila* peripheral nervous system (Yamamoto et al., 2014). A secondary mosaic screen was then performed in this mutant collection by creating mutant clones in the antenna to identify mutants with an abnormal Johnston's organ (Li et al., 2016). As discussed in previous sections, genes implicated in hearing in these *Drosophila* screens include those known to cause hearing loss in humans or mice. The convenience of *Drosophila* as a model organism, including its short life span and armory of genetic tools, makes it an attractive system to investigate the function of genes involved in hearing. Historically, overexpression experiments have been used to test gene function in *Drosophila*, either using the relevant fly gene or a conserved mammalian homolog. However, it is not always possible to unambiguously identify mammalian homologs of *Drosophila* genes based on sequence alone. Moreover, these approaches are not necessarily able to dissect why particular human gene variants are pathogenic, especially in the case of missense mutations. In addition, overexpressing a gene or gene variant at nonphysiological levels can lead to experimental artifacts. In recent years, advances in recombination-mediated cassette exchange (RMCE; Box 1) technology and the ability to perform homologous recombination in *Drosophila* using the CRISPR-Cas9 system (Box 1) have allowed researchers to rapidly test the function of human genes in *Drosophila* and to rapidly assay the effects of potentially pathogenic human variants far more quickly than in mice. In this section, we describe the components of this technology, which is summarized in Fig. 5.

### Gene trap cassette integration and exchange

An identified ortholog of a human or mouse gene is a prerequisite for functional testing in *Drosophila*. Although not all candidate disease genes will have invertebrate orthologs, ∼60% of the protein-coding genes in the *Drosophila* genome are conserved in mammals, and *Drosophila* orthologs have been identified for about two-thirds of human disease genes (Chien et al., 2002; Hu et al., 2011; Reiter and Bier, 2002; Spradling et al., 2006). Numerous *Drosophila* Minos-mediated integration cassette (MiMIC) lines are available (Venken et al., 2011) that can be further modified using RMCE (Nagarkar-Jaiswal et al., 2015a,b). Using this technique, an artificial exon that serves as a gene trap – such as a GAL4 Trojan exon cassette (Diao et al., 2015) – can be inserted into a MiMIC line to create a premature termination signal that disrupts the function of the endogenous gene. If MiMIC lines are not available or suitable for a particular gene, it is now possible to insert MiMIC-like cassettes in *Drosophila* genes using CRISPR-mediated homology-directed repair (Port et al., 2014; Ren et al., 2013).

### Green fluorescent protein tagging to characterize expression and function

Artificial exons can be created to have flanking sites that facilitate RMCE (Nagarkar-Jaiswal et al., 2015a,b; Venken et al., 2011). These exons allow fluorescent tags to be introduced into a gene of interest to reveal its cellular and subcellular expression pattern (Fig. 5). This approach has been used to modify hundreds of *Drosophila* genes, providing detailed expression data and with ∼75% of the genes retaining their function after incorporation of the internal exon-encoding green fluorescent protein (GFP), as shown by their ability to rescue null alleles (Nagarkar-Jaiswal et al., 2015b). To date, nearly 10,000 *Drosophila* genes have been GFP tagged in this way (Kanca et al., 2017). These lines also allow gene and protein function to be tested through the targeting of the GFP-encoding exon with RNA interference (RNAi) approaches (Neumuller et al., 2012; Pastor-Pareja and Xu, 2011). In the adult fly, however, the stability of some proteins in postmitotic adult cells can preclude efficient RNAi knockdown. To address this, a genetic system known as the deGradFP system can be used to directly degrade the GFP-tagged protein by using a modified form of the ubiquitin-proteasome pathway to specifically target and degrade GFP-tagged proteins (Caussinus et al., 2012, 2013) (Fig. 5).

### Testing auditory function in *Drosophila*

*Drosophila* mutants can be assessed for auditory function in several ways. In adult flies, a sound-evoked compound action potential can be recorded from the antennal nerve (Eberl et al., 2000; Todi et al., 2008), although this method is less sensitive if small clones of mutant cells are being assayed in an otherwise wild-type background (Li et al., 2016). Behavioral assays can also be used to detect hearing in response to stimuli such as mating songs (Eberl et al., 1997), or the response of flies to the force of gravity (Kamikouchi et al., 2009), although, once again, these suffer from a limited sensitivity. It is also possible to measure active movement of the antenna by laser vibrometry and to record the activity of single giant neurons in the *Drosophila* brain (Kamikouchi et al., 2009; Tootoonian et al., 2012). Finally, it is possible to examine the morphology of Johnston's organ in different mutants using an array of antibodies and GAL4 lines (Box 1) that express fluorescent proteins in the cellular components of scolopidia.

### Testing mammalian homologs and pathogenic variants

Having identified and characterized the function of *Drosophila* orthologs of mammalian genes in hearing, it is possible to test their functional conservation by rescuing a *Drosophila* mutation with one of its human or mouse orthologs. The use of GAL4-Trojan gene traps (Fig. 5) is particularly useful in this regard. In this approach, the GAL4 transcription factor is targeted to an endogenous *Drosophila* exon. By fusing the GAL4 sequence to a T2A peptide-coding sequence, the endogenous protein begins to be translated, but is then truncated and GAL4 protein is expressed instead. This transcriptional activator then activates the expression of a mammalian complementary DNA (cDNA) of interest under the control of a UAS promoter from a separate locus in the *Drosophila* genome (Fig. 5). Thus, it is possible to inactivate a *Drosophila* gene and simultaneously assay whether a mammalian homolog can functionally rescue the inactivated gene. Moreover, once it has been established that a mammalian cDNA can rescue its *Drosophila* ortholog, it is then possible to test variants of the mammalian gene to determine whether they are pathogenic, and if so, to evaluate the cellular or biochemical consequences of such pathogenic variants (Bellen and Yamamoto, 2015). The function of potentially dominant variants can also be assessed in this way by overexpressing the variant of interest in Johnston's organ to see if it elicits a phenotype. For example, pathogenic variants of *Myh9* known to cause deafness in humans also cause morphological defects in scolopidia when overexpressed in Johnston's organ (Li et al., 2016).

The GAL4-Trojan system has several advantages over conventional methods to test gene function by overexpression. First, it can test the function of a mammalian gene in the context of a null mutation for its *Drosophila* counterpart. Second, targeting the GAL4 cassette to the *Drosophila* gene of interest significantly increases the likelihood that the GAL4 activator will be expressed in the same spatial and temporal pattern as the endogenous *Drosophila* gene. Finally, the incorporation of DNA sequences in the Trojan cassette that permit RMCE allows the locus to be modified further with little effort. Although these advances are significant, they still have limitations. For example, this approach is only feasible if clear gene homologs can be identified between *Drosophila* and mammals. Moreover, although expression of the GAL4 transactivator might be faithful, the UAS promoter might drive expression of the mammalian gene at levels greater than those of the endogenous *Drosophila* gene.

## Conclusion

The field of genetic hearing loss research has advanced significantly in the last several decades, but three challenges and opportunities remain. First, it is likely that many new genes that regulate hearing await discovery. The study of extended families, particularly those that feature consanguineous marriages, has revealed new deafness gene candidates, but following up these new candidates is hampered by the limited availability of these large families in restricted regions of the world. The National Institutes of Health (NIH)-funded Undiagnosed Diseases Network (http://undiagnosed.hms.harvard.edu) seeks to find possible genetic etiologies for rare human diseases (Wangler et al., 2017), some of which include hearing loss, and it is likely that this initiative will reveal new forms of syndromic hearing loss. In addition to the ongoing study of human families with hereditary deafness (Smith et al., 2014), there is currently a worldwide effort to create, characterize and curate mutations in every mouse gene coordinated through the International Mouse Phenotyping Consortium (IMPC) (www.mousephenotype.org), with the US effort coordinated through an NIH-funded Knockout Mouse Project (www.komp.org). Because auditory testing is part of the standard IMPC phenotyping pipeline, it is likely that many new mouse genes will be identified that can cause hearing loss in homozygous or heterozygous mutants, and this might support the identification of human variants that also cause hearing loss. Mutagenesis-based screens in mice have also revealed new genes responsible for hearing loss and age-related hearing loss (Hrabe de Angelis et al., 2000; Nolan et al., 2000; Potter et al., 2016).

Although over 100 deafness genes have been identified, a second challenge is to understand the function of these genes in hearing. While the cellular and biochemical functions of some of these genes have been extensively characterized, many other known deafness genes remain poorly characterized. Moreover, the pathogenic basis of many human deafness gene variants await elucidation. In this Review, we have suggested that the homologies between insect and mammals, in terms of the genes involved in hearing organ development and function, make *Drosophila* an attractive system for assaying the function of deafness-associated gene variants. We believe that the technology described in this Review will make it possible to perform medium- and high-throughput screens of candidate mammalian genes and known deafness-associated genes in *Drosophila*, in addition to identifying novel genes that are essential for hearing in *Drosophila* that might have mammalian homologs.

Once the pathogenic mechanisms of human deafness gene variants have been established, the final challenge is to develop therapies for particular forms of deafness that do not rely on hearing aids or cochlear implants. As with other forms of genetic disease, it is possible to improve function by pharmacological approaches that, to name but three, stabilize protein complexes, enhance the function of the affected protein(s) or proteins acting downstream of the affected proteins, and slow the aggregation of pathogenic protein variants. Once again, we suggest that *Drosophila* might offer a promising platform in which to rapidly screen for compounds or signaling pathways that improve auditory function in animals bearing pathogenic hearing gene variants.
